# From meekness to influence: rethinking oral health advocacy

**DOI:** 10.1038/s41415-026-9647-1

**Published:** 2026-09-11

**Authors:** Habib Benzian, Sudeshni Naidoo

**Affiliations:** 41415740821001 University of the Western Cape, Cape Town, South Africa; Chulalongkorn University, Bangkok, Thailand; Sydney Dental School, Faculty of Medicine and Health, The University of Sydney, Sydney, Australia; 41415740821002 WHO Collaborating Center for Oral Health, Department of Community Dentistry, University of the Western Cape, Cape Town, South Africa

## Abstract

Oral health remains a soft voice in loud global health spaces. Other healthcare fields have secured coalitions, goals, governance, and entitlements; oral health often settles for symbolic inclusion and pilot projects. This paper uses ‘meekness' as a metaphor for a structural posture of oral health advocacy marked by caution, deference, and lack of political leverage. Meekness is expressed across four domains: professional voice, institutional platforms, academic culture, and civic positioning. They create an architecture that privileges visibility over influence. Professional associations dominate public discourse but are closely tied to clinical and commercial interests. Weak civil society and groupspeak rhetoric reinforce cautious advocacy, while charity and awareness campaigns substitute for structural reform. Academic incentives discourage outward-facing scholarship and public intellectual leadership, muting analytic and civic pressure. Together, these factors sustain marginality in universal health coverage despite a large disease burden and strong evidence for prevention. The paper outlines what full recognition would entail: defined essential services, stable financing, primary healthcare-based delivery, diversified workforce models, and regulatory action on commercial determinants. Moving from meekness to power requires explicit demands for benefits, budgets, and rules, stronger civic alliances, and academic cultures that reward public reasoning as part of systemic change.

## Key points

- Oral health advocacy is shaped by structural meekness: symbolic inclusion without systemic influence across professional, institutional, academic, and civic domains.
- Meekness sustains marginality in health policy through weak civic voice, cautious rhetoric, and substitution of charity for structural reform.
- Shifting to power requires explicit demands for benefits, budgets, and rules, alongside stronger civic alliances and public-facing academic leadership.

## Introduction

Oral health remains a soft voice in loud global health spaces. Where other global health communities have built broad coalitions, set goals, created governance structures, and secured entitlements, oral health often settles for polite invitations to side events, working groups, and pilot projects. In this analysis, ‘meekness' is not a personality trait, but a metaphor for a structural posture of the oral health field within global health advocacy. Meekness here does not describe intent, effort, or professional commitment, but a patterned relationship to power in which participation and visibility are weakly coupled to leverage over rules, budgets, and binding decisions. The term, though rarely used in policy discourse, aptly captures a pattern of symbolic presence without systemic influence, marked by deference, caution, and reluctance to assert power.

Meekness in oral health advocacy persists across four domains: the professional voice, institutional platforms, academic culture, and civic positioning. Together, they form a recurring architecture of meek advocacy that favours politeness over precision, and visibility over power. For decades, the primary voice in oral health advocacy has come almost entirely from within dentistry and from academic actors rooted in clinical work, rather than in policy or health system reform. These actors are often structurally tied to the very arrangements they would need to challenge. This helps explain the persistent marginality of oral health in universal health coverage, chronic under-financing, and high out-of-pocket spending, despite a massive burden and strong evidence for prevention. The issue examined in this paper is therefore not the speed of change, but the absence of mechanisms that reliably convert policy recognition and technical consensus into durable authority and system-level action.

Moving from meekness to power requires a sharper political strategy, clearer vision, and explicit demands for benefits, budgets, and rules.^1^^,^^2^^,^^3^ This argument is made against the backdrop of unprecedented normative and policy progress for oral health in recent years, marking a qualitative shift in global recognition compared to earlier decades. In this period, the World Health Organization has led global oral health norm-setting and agenda formation, with many other organisations and stakeholders subsequently aligning their strategies and commitments.

Yet unlike milestones in human immunodeficiency virus (HIV), tobacco control, or maternal health, the contemporary global oral health policy architecture established since 2021 was not driven by organised civic demand. It emerged from academic and technical processes, like the *Lancet* series on oral health.^1^^,^^4^^,^^5^^,^^6^^,^^7^^,^^8^ Policy developed in the absence of sustained external pressure rarely secures serious financing or public accountability. It may deliver visibility, but often without the power to transform.

As a result, even as recent resolutions, global strategies, commissions, and high-level declarations have produced a dense policy architecture for oral health, often giving rise to important but exceptional examples of progress, a central question remains: why has this architecture delivered recognition without corresponding sustained mainstream authority, financing, or enforceable entitlements? These advances demonstrate feasibility and potential, but their exceptional nature also illustrates why accumulating evidence and isolated progress have not translated into system-wide access, protection, or durable reductions in inequality.

In this analysis, advocacy is understood as the act of articulating and advancing specific demands through political strategy, public accountability, and institutional pressure.^9^ Meekness, by contrast, retreats from this mode. It privileges symbolic gestures and technical consensus. It seeks acceptance rather than leverage, favours polite requests over binding claims, and rarely names precise entitlements, financing mechanisms, or binding governance instruments of change.

## Professional voice, groupspeak, and the missing public

In many health systems, oral health sits at the margins of government planning and decision processes, always at risk of being overlooked or excluded entirely. The field's public voice reflects a pattern of institutional meekness, with a tendency to accommodate prevailing structures rather than assert alternative priorities or challenge underlying imbalances. While such caution may preserve access and relationships, it also limits the field's capacity to shift its position from the periphery to the core of health system design. Instead of demanding full integration and recognition at parity with other priorities, advocacy often pleads for inclusion at the edges through pilots and incremental adjustments. This defensive posture, while rational from the standpoint of institutional survival, reflects a deeper entrenchment of meekness. It exchanges long-term structural influence for short-term accommodation, reinforcing the peripheral status of oral health in broader health agendas. Yet, a profession holds a privileged status only as long as it serves the public first.^10^ When the advocacy agenda places economic, commodity, or cosmetic interests ahead of population need, credibility erodes.^11^^,^^12^

Because independent civil society in oral health is weak, professional associations have become the public voice by default. However, that voice often carries the imprint of institutional meekness, challenged by disentangling its clinical obligations from its commercial interests.^13^ Dentistry addresses urgent needs such as pain, infection, and function, but it is also embedded in consumer markets for elective and appearance-oriented services. Aesthetics are by no means inherently illegitimate. The relevant distinction is needs-fulfilment versus needs-creation. When professional narratives and marketing blur health with economics, or the medicalisation of normal variation, advocacy risks shifting from a voice for the public and becomes an echo of the market.^14^^,^^15^

Internal dynamics make the problem worse. In many settings, groupspeak produces a unified and cautious public line. The vocabulary is familiar: oral health is neglected, integration is needed, and prevention is good.^16^ These formulas reflect a meek rhetorical mode, one that avoids internal disagreement and sidesteps politically sensitive trade-offs, including complicity with private, high-cost models of care. Prevention, in particular, is often asserted without being defined, described, or operationalised. Its invocation signals virtue but lacks the specificity required to shape budgets, rules or services. The result is a framing of neglect without a concrete vision of what full integration would mean:

- Which services are essential and guaranteed
- How financing should flow to reduce catastrophic spending
- What scopes, team models, and primary care protocols are required
- Which governance and accountability mechanisms will bind commitments.

Without such specificity, advocacy remains a description of the litany of problems rather than an urgent demand for change.^17^ Raising these questions would expose intra-professional trade-offs, which groupspeak seeks to suppress.

The meekness of oral health advocacy is not only professional, but also civic and reinforced by the absence of strong patient movements.^18^ Oral disease leaves visible signs such as missing teeth and untreated decay. These are often portrayed as personal failure rather than symptoms of systemic neglect.^19^ This framing fosters shame and self-blame, weakening public motivation to organise and engage in civic discourse. Unlike HIV or cancer, the oral health fraternity lacks broad-based coalitions that make inaction politically costly. In this vacuum, where civic pressure is weak and the professional voice remains cautious, alternative forms of symbolic social responsibility often occupy the space, generating positive publicity and reinforcing professional ethical branding.

## Substitution ethics and weak civic leverage

These symbolic social responsibilities often take the form of charitable action. Short-term volunteer missions and free clinics provide visible relief and compelling stories. But they also allow professionals to display benevolence while leaving unjust financing and arrangements for access to care unchanged. Temporary pop-up clinics can prioritise provider values, routinise gatekeeping, and displace structural reform by converting moral urgency into a public relations performance. Charity can and should illuminate need, but it cannot dismiss a public obligation to guarantee access.^20^

Awareness campaigns often mirror the same meek logic. They are safe, media-friendly, and consensus-oriented, but rarely shift outcomes unless they directly target entitlements, budgets or regulatory change within the health system. Awareness without access does not empower, it frustrates. It can even widen inequities by stimulating demand that only the insured and affluent can afford. Health education and health literacy initiatives often accompany such campaigns, but their impact remains limited without structural backing. The evidence for standalone effectiveness is weak, especially in settings where affordability, service availability, and regulatory context are the true barriers to access.^21^^,^^22^^,^^23^

A credible alternative is to translate awareness into specific and verifiable demands that name essential services for universal coverage, identify multi-year budget lines with implementation targets, and revise the rules that govern delivery and the commodity environments that drive disease. If a campaign cannot point to the line item, the legal text, or the practice rule it will change, it relies on sentiment rather than strategy. Success must be measured in coverage, protection from catastrophic dental spending, waiting times, and the distribution of these gains. It is not measured in social media impressions or web traffic.

## Framing choices sustain meekness

Several common discursive and strategic frames help explain why the oral health field continues to operate with a posture of policy meekness.^24^ The securitisation frame, which has worked powerfully for infectious diseases, is underutilised. Risks linked to free sugars and to catastrophic dental expenditure are routinely individualised rather than framed as systemic threats transmitted through commercial vectors and shaped by policy design.^25^ This reluctance to frame oral health as a matter of collective security reflects a deeper hesitancy to confront entrenched power. Dental associations and academic advocates have been too meek to engage with regulatory actions that challenge the interests of influential corporate conglomerates.

The moralisation frame is common but remains inconsistent. Appeals to essentiality, to the emblematic power of the smile, and to value-based care often coexist with narratives that drift into behavioural blame. As a result, moral claims rarely anchor a broader social justice strategy.^26^ Technification frames dominate the discourse, portraying oral diseases as technical problems solvable through clinical interventions. While the rhetoric is strong, operational models and impact evidence remain limited. Economic framing has gained traction, but health economics capacity and cost data remain too limited to persuade ministries of finance at scale.^27^^,^^28^ These framing habits sustain a kind of strategic meekness: advocacy that seeks consensus and visibility without invoking pressure, leverage, or contestation.

A second domain of meekness lies in the platforms through which advocacy is pursued. Single-issue campaigns have delivered breakthroughs for emblematic conditions such as noma.^29^^,^^30^ Diagonal approaches that integrate issue-specific efforts into broader system strengthening are often recommended, but rarely operationalised in oral health.^31^ Despite frequent references to integration, practical models remain scarce, fragmented, and under-evaluated. A pragmatic path is to render diagonal strategies concrete.^32^ This would require more than rhetorical alignment. It requires explicit benefit definitions for priority conditions, clear care pathways in primary care, defined roles for team members including mid-level providers, and fiscal mechanisms such as earmarked taxes on sugar-sweetened beverages tied to prevention and coverage. This is not about labels. It is about structural specificity: who does what, where, and with which resources.

## Professional socialisation and the trap of incrementalism

Professional education often introduces inequity through short-term outreach or humanitarian placements. These experiences can be humane and formative. But they also socialise future providers into episodic benevolence rather than structural advocacy.^33^ Community-based dental education and volunteer events can valorise the exceptional, gamify participation, and centre provider experience with limited evidence of durable shifts toward careers that expand equitable access. The result is a pipeline that is fluent in technique and charity but faltering in justice.

This socialisation feeds a broader pattern of incrementalism rooted in institutional meekness. For decades, the profession has negotiated for crumbs. Pilot projects, demonstrations, and mentions in strategies are offered and presented as signs of progress. The outcome, however, is a stagnant equilibrium, visibility without financing, policy language without entitlements, technical integration without political power. Meanwhile, people pay the price in pain, disability, and financial hardship. Unless advocacy engages with the real levers such as financing flows, benefit design, workforce models, and regulation of commercial determinants, oral health will remain peripheral.^1^^,^^34^

## Silenced scholarship and the absence of public voice

The thin bench in academic advocacy is not just a capacity gap. It is a conduit through which meekness returns to the centre of the field. When universities reward clinical trials, laboratory output and high-impact publications over policy craft and public engagement, scholars learn to keep their voice narrow and their horizon short. When contracts come from soft money and tenure is elusive, the safest path is to publish within disciplinary lanes and avoid political entanglement.^35^^,^^36^ In this environment, the academic voice becomes cautious, episodic, and inward looking. That restrained voice reinforces professional groupspeak and leaves civil society without an empathetic partner that can translate concern into instruments the system must act on.

The field also lacks public intellectuals who can speak across audiences with authority and moral clarity.^37^ In other health domains, public intellectuals perform a bridging role. They condense evidence without distortion, name the choices that matter, and keep issues on the agenda when attention drifts. They do not only write papers. They frame debates, testify before committees, convene unlikely allies, and propose texts that can be adopted in law or regulation. Oral health has only a few figures who carry that civic mandate. The absence is not a matter of individual courage alone. It reflects how institutions define and value scholarship, the scarcity of endowed platforms for policy work, and the limited availability of full-time funding for outward-facing roles.

In the absence of such public voices, the default becomes quietism. The field speaks to itself, rarely to government, finance ministries or political platforms, and almost never to the public whose support can shift the politics of coverage. In this setting, meekness no longer looks like failure. It looks like professionalism. Asking for awareness and direction seems prudent, avoiding conflict seems strategic. The missing public voice from universities is not an ornamental absence, but a structural condition that helps understand why oral health remains peripheral even when the evidence is strong.

A different academic attitude and culture would break this circuit. It would cultivate scholars who are at ease in committee rooms and on broadcast media, who can craft benefit definitions and also explain them to parent associations and labour unions, who monitor policy calendars and anticipate openings rather than react to them. It would treat public reasoning as part of academic merit, not as extracurricular activity. With such a collective resolve, the field could pair professional leadership with civic persuasion and watchdog functions and move from a survival mode to an agenda-setting, catalytic role.

## The missing vision: what full integration would mean

One of the most striking features of oral health advocacy is the absence of a clear and actionable vision of full integration. The language of neglect is familiar. But far fewer actors can describe what an integrated, financed, accountable oral health system at parity with other priorities would look like. The lack of such a vision is not merely an oversight; it reflects the broader caution that defines much of the field's advocacy.

A credible vision would define essential services as a guaranteed part of universal coverage. It would specify how these services are financed and delivered, and how households are protected from catastrophic out-of-pocket spending. It would set out workforce models that span the entire team with clearly defined roles, scopes of practice, and quality assurance so that coverage expands safely. These cadres would collaborate with other health care professionals to embed oral health in general health. Integration would be visible in primary care through shared records, referral systems, prevention protocols, and team-based practice, rather than being siloed in specialist clinics. It would also include governance and accountability: budgets with named lines and execution targets, and monitoring systems that track coverage and financial protection with public reporting. Commercial determinants would be addressed through regulation of product environments and fiscal tools such as taxes on sugary drinks, with transparent reinvestment in prevention. These building blocks are not aspirational; they are routine features of other health system functions. Oral health remains an exception largely because political and institutional mechanisms to deliver them are weak or absent.

A practical entry point already exists. The 6 × 6 approach expands the current 5 × 5 non-communicable disease (NCD) framework to include oral diseases and sugars.^38^^,^^39^ It provides a concise, communicable structure to position oral health squarely within high-level NCD strategies, making it easier to secure political attention, align with financing streams, and shape benefit packages. By placing oral diseases alongside other major NCDs, and sugars alongside the most powerful risk factors, the 6 × 6 framework offers a strategic lever for integration and influence.

Such a new oral health advocacy vision should be judged by a few demanding criteria. Does it prioritise necessary services before elective interventions, ensuring that prevention, pain and infection control, tooth preservation, and essential services are central? Does it treat equity as a binding frame by testing effects on access, affordability, and availability? Is it insulated from commercial inducement, so that public messaging aligns with health need rather than consumer marketing? Does it support role diversification and team-based models where these expand access safely? Does it focus on structural change, using charity and awareness only as entry points for deeper policy reform? When such criteria are met, advocacy moves from description to design.

## The way forward: from meekness to power

The dynamics explored in this paper across voice, platforms, scholarship, and civic stance are summarised in Table 1. These typologies are not academic abstractions; they describe real constraints that must be named and dismantled.

**Table 1:** Domains of meekness in oral health advocacy

**Domain**	**Sub-domain**	**Expression of meekness**	**Negative impact on advocacy**	**Shift required**
1. Voice	Language and messaging	Soft demands, vague appeals	Mutes urgency	Strategic clarity, public engagement
	Internal discourse	Groupthink, avoidance of conflict	Reinforces status quo	Honest debate, intra-professional realism
2. Platforms	Integration models	Pilots over policy, fragmented services	No binding commitments	Defined pathways, benefit clarity
	Policy positioning	Inclusion without leverage	Symbolic presence	Demand entitlements and influence
3. Scholarship	Knowledge production	Apolitical, inward-facing scholarship	Depoliticised research	Civic-oriented, policy-facing inquiry
	Intellectual leadership	Absence of public reasoning or figures	No analytic partner for civil society	Enable and elevate public intellectual roles
4. Civic stance	Ethical posture	Charity over rights, aesthetics before equity	Moral substitution	Rights-based framing, essential care
	Patient and civic engagement	Internalised stigma, weak mobilisation	Low political pressure	Amplify patient voice, build coalitions

The analysis highlights that meekness has preserved autonomy and status, but only by accepting marginality. Meekness allows for a seat at the table without leverage over the menu. But meekness is no longer a neutral option because it entrenches inequities, silences patients, and protects markets rather than people. Moving beyond meekness means replacing the language of neglect with a concrete design for integration. Benefits, budgets, roles, and rules must be named with precision. Groupspeak must give way to honest internal debate as the price of progress. Partnerships with civic actors beyond the profession must be built. Speaking softly and asking politely for awareness, inclusion, recognition, or a seat at the table is no longer enough. This is not an argument for oral health to displace other health priorities, but for it to be taken seriously at parity within shared reform agendas where decisions about coverage, financing, and accountability are made. The profession must demand benefits, budgets, and binding rules and be willing to fight for them.

Meekness is not only a habit of professional associations. It is also a predictable product of academic incentives that sideline public intellectual work and reward technical refinement over political engagement. Recent academic contributions illustrate both the progress achieved and the limits of prevailing advocacy approaches.^40^^,^^41^ These contributions articulate increasingly sophisticated agendas, emphasising coalition-building, shared terminology, sustainability, planetary and One Health linkages, and a clear shift away from narrow biomedical models. They reflect genuine conceptual maturation and a sustained effort to reposition oral health within wider health, environmental, and societal debates.

Yet they also reveal a persistent analytic constraint. Many such approaches rely, implicitly or explicitly, on linear logics of change in which evidence generation, consensus formation, and normative alignment are assumed to translate into political priority and system reform. In doing so, they tend to underplay the political economy of oral health: entrenched institutional arrangements, professional interests, market dynamics, and distributional conflicts that shape which evidence gains traction, how agendas are filtered, and which reforms remain feasible.

If even this level of conceptual sophistication, evidence, and normative alignment fails to generate durable authority, financing, and enforceable entitlements, then the constraint is not intellectual or technical, but structural and political. As long as this economy of voice remains unchanged, oral health will bring strong evidence and carefully framed claims into arenas where power responds primarily to those who speak clearly, publicly, and with consequence. This analysis may seem sobering, but it is not intended to discredit the field. Many advocates are already advancing bold strategies and moving the agenda forward. The critique is directed instead at outdated default assumptions that still shape much of the oral health advocacy field's vocabulary and practice. Reverting to health education, literacy and awareness as solutions for oral diseases is no longer credible. These approaches may assist, but they cannot substitute for structural change.

The shift from meekness to power is therefore not just a strategic choice. It is an imperative for political maturity in a field that can no longer afford to whisper at the periphery of health reform.
